# The *Fusarium graminearum* FGSG_03624 Xylanase Enhances Plant Immunity and Increases Resistance against Bacterial and Fungal Pathogens

**DOI:** 10.3390/ijms221910811

**Published:** 2021-10-06

**Authors:** Silvio Tundo, Maria Chiara Paccanaro, Valentina Bigini, Daniel V. Savatin, Franco Faoro, Francesco Favaron, Luca Sella

**Affiliations:** 1 Department of Land, Environment, Agriculture and Forestry (TESAF), University of Padova, Viale dell’Università 16, 35020 Legnaro, PD, Italy; silvio.tundo@unipd.it (S.T.); mariachiara.paccanaro@hotmail.it (M.C.P.); Francesco.favaron@unipd.it (F.F.); 2 Department of Agriculture and Forest Sciences (DAFNE), University of Tuscia, Via S. Camillo de Lellis snc, 01100 Viterbo, VT, Italy; valentina.bigini@unitus.it (V.B.); daniel.savatin@unitus.it (D.V.S.); 3 Department of Agricultural and Environmental Sciences, University of Milano, Via Celoria 2, 20133 Milano, MI, Italy; franco.faoro@unimi.it

**Keywords:** cell wall degrading enzymes (CWDEs), wheat, callose, *PR1*, *Pseudomonas syringae*, *Botrytis cinerea*

## Abstract

Fungal enzymes degrading the plant cell wall, such as xylanases, can activate plant immune responses. The *Fusarium graminearum* FGSG_03624 xylanase, previously shown to elicit necrosis and hydrogen peroxide accumulation in wheat, was investigated for its ability to induce disease resistance. To this aim, we transiently and constitutively expressed an enzymatically inactive form of FGSG_03624 in tobacco and *Arabidopsis*, respectively. The plants were challenged with *Pseudomonas syringae* pv. *tabaci* or pv. *maculicola* and *Botrytis cinerea*. Symptom reduction by the bacterium was evident, while no reduction was observed after *B. cinerea* inoculation. Compared to the control, the presence of the xylanase gene in transgenic *Arabidopsis* plants did not alter the basal expression of a set of defense-related genes, and, after the *P. syringae* inoculation, a prolonged PR1 expression was detected. *F. graminearum* inoculation experiments of durum wheat spikes exogenously treated with the FGSG_03624 xylanase highlighted a reduction of symptoms in the early phases of infection and a lower fungal biomass accumulation than in the control. Besides, callose deposition was detected in infected spikes previously treated with the xylanase and not in infected control plants. In conclusion, our results highlight the ability of FGSG_03624 to enhance plant immunity, thus decreasing disease severity.

## 1. Introduction

Fusarium Head Blight (FHB) is a global crop disease with a great economic impact on the cereal industry [1,2]. The disease reduces grain yield and quality of products for the contamination by mycotoxins harmful to humans and animals [3,4]. Different Fusarium species are associated with the disease, but *Fusarium graminearum* is the most common causal agent in cereal crops, especially wheat [5].

In the early stages of wheat spike infection, *F. graminearum* produces a wide range of cell wall-degrading enzymes (CWDE) such as cellulases, pectinases and xylanases [6]. The timing of secretion of these enzymes suggests that CWDEs facilitate rapid colonization of spike tissues through the degradation of the cell wall, which represents the first physical barrier against pathogens. The importance of these enzymes in the infection process is also supported by the evidence that the overexpression in transgenic wheat plants of protein inhibitors of CWDEs resulted in reduced symptoms of FHB [7,8,9,10].

Among CWDEs, endo-β-1,4-xylanases (xylanases; EC 3.2.1.8) are responsible for the degradation of xylan, the main component of cell walls of commelinids monocot plants [11]. According to their catalytic activities and structure, xylanases are mainly grouped into GH10 and GH11 (glycoside hydrolase, CAZy classification) families, with some members belonging to other GH families [12].

Early work on fungal xylanases demonstrated that, besides the enzymatic activity, some xylanases could also induce programmed cell death (PCD) and activate the plant immune responses, as shown for EIX and Xyn11A xylanases of *Trichoderma viride* and *Botrytis cinerea*, respectively [13,14].

For example, the xylanase Xyn11a of *B. cinerea* contributes to virulence on grape and tomatoe with its necrotizing activity and not its enzymatic activity [14]. Interestingly, a short 25-residues peptide from that xylanase determines the effects caused in plants by the entire protein, including necrosis and activation of plant defense responses [15]. Another *B. cinerea* xylanase named BcXyl1 has been demonstrated to be a virulence factor [16] and, similarly to Xyn11a, showed the ability to induce cell death and plant immunity in several plants independently from its enzymatic activity. This capacity has been suggested to be related to the binding to a putative plant receptor, as shown for *T. viride* EIX xylanase that interacts with a plasma membrane receptor protein essential for cell death elicitation [17].

In wheat, *F. graminearum* expresses several xylanases during spike infection [18], including the xylanases FGSG_03624, FGSG_10999 and FGSG_11487 that induce necrosis in wheat tissues [18,19]. As observed with *B. cinerea*, these *F. graminearum* xylanases also induce necrosis unrelated to the enzymatic activity. Noteworthy, the GH11 xylanase FGSG_03624 shares with the *B. cinerea* Xyn11a two peptide stretches, each of four amino acids, within the 25 amino acids region of Xyn11a indicated as essential to activate host defense responses [15].

Despite the enzymatic and necrotizing activities of xylanases, disruption experiments carried out on *FGSG_03624* as well on the major xylanase gene regulator *Xyr1* in *F. graminearum* do not support a significant contribution for xylanases in FHB [18,20] unless other CWDEs are also simultaneously disrupted [21]. In contrast, the deletion of the *FGSG_10999* encoding gene suggests a contribution of this xylanase in fungal virulence [22,23]. In addition, the observation that the overexpression of TAXI-III, a xylanase inhibitor of GH-11 xylanases, in wheat plants’ delayed *F. graminearum* infection supports a contribution of the xylanase activity in the progression of FHB [8].

Since molecules inducing PCD near the treatment site are often associated with activation of immune responses [24], we have investigated if the *F. graminearum* FGSG_03624 xylanase (hereafter named as Xyl) could modulate plant immunity acting as a PAMP and increasing plant resistance to pathogens. To this aim, we preliminarily treated Arabidopsis plants with the purified Xyl to verify the capacity of this xylanase to increase resistance against two commonly used pathogens of this model plant, the fungus *B. cinerea* and the bacterium *Pseudomonas syringae*. Then, the resistance and immunity responses were determined in tobacco and Arabidopsis plants transiently or constitutively expressing the xylanase gene, respectively. In these latter cases, site-directed mutagenesis was performed to inactivate the enzyme’s catalytic site to exclude side effects of the protein on the plant cell wall. Finally, we verified the possibility of treating durum wheat plants with xylanase to increase resistance against *F. graminearum*.

## 2. Results

### 2.1. The Treatment with Xyl Increases Arabidopsis Resistance against *P. syringae* pv. *maculicola*

**Figure 1:**
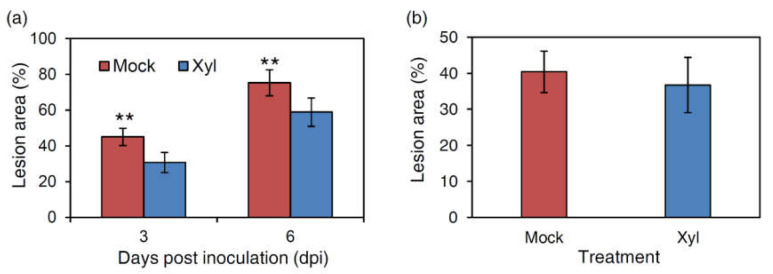
Infection experiments of Arabidopsis leaves with (**a**) *Pseudomonas syringae* pv. *maculicola* or (**b**) *Botrytis cinerea* after exogenous treatment with the FGSG_03624 xylanase. Leaves treated with water were used as negative control (mock). Symptoms were evaluated at 3 and 6 days post-infection (dpi) for *P. syringae* and at 2 dpi for *B. cinerea*. Data (percentage of the infected area on total leaf area) represent the average ± mean standard error (SE, indicated by bars) of at least three independent experiments. ** indicate significant differences at *p* < 0.01.

To investigate a possible induction of disease resistance, the previously characterized and heterologously expressed Xyl [18] was sprayed on Arabidopsis plants, and, 4 days after the treatment, the plants were inoculated with *P. syringae* pv. *maculicola* or *B. cinerea*. Compared to control leaves sprayed with water only (mock), in the xylanase treated leaves, the size of the bacterial-induced spots at 3 dpi and 6 dpi was significantly reduced by 25% and 20%, respectively (Figure 1a). Differently, the average size of *B. cinerea* lesions in the Xyl treated plants was comparable to that of control plants (Figure 1b).

### 2.2. Transient Expression of an Inactive Version of the Xylanase (mXyl) in Tobacco Leaves by Agro-Infiltration Increases Resistance to *P. syringae* pv. *tabaci* but Not to *B. cinerea*

**Figure 2:**
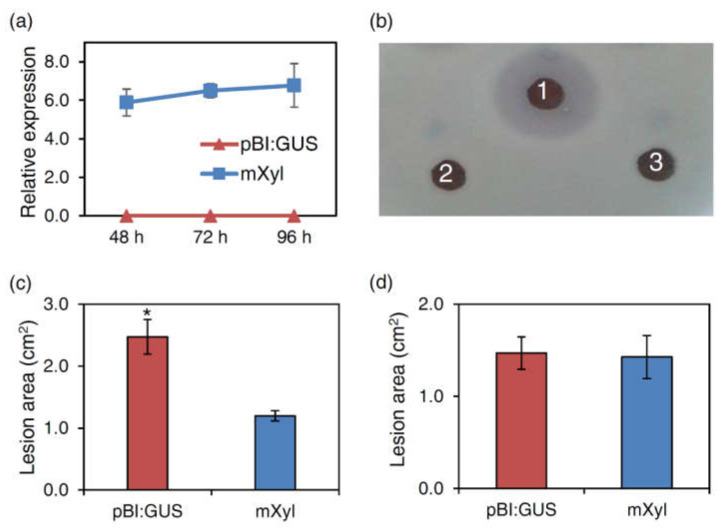
Characterization of tobacco cv. SR1 plants agro-infiltrated with the enzymatically inactive FGSG_03624 xylanase (mXyl): (**a**) Relative expression of the *mXyl* transcript in mXyl and pBI:GUS expressing plants determined by qPCR. Data represent the average ± mean standard deviation (SD, indicated by bars) of two independent experiments; (**b**) Radial gel diffusion assay to quantify the xylanase activity: halo produced by 1.5 U of the *Fusarium graminearum* FGSG_03624 xylanase (1); total protein extract from tobacco plants agro-infiltrated with *mXyl* (2) or pBI-GUS as negative control (3). (**c**) Lesion area produced by *Pseudomonas syringae* pv. *tabaci* on tobacco leaves expressing mXyl compared to control leaves agro-infiltrated with pBI:GUS at 4 dpi. Bars indicate the standard error (SE) calculated from three independent infection experiments. Statistical analysis was performed by applying Student’s *t*-test. * indicates significant difference at *p* < 0.05; (**d**) Lesion area produced by *Botrytis cinerea* on tobacco leaves expressing mXyl or pBI:GUS at 3 dpi. Bars indicate the standard error (SE) calculated from three independent infection experiments.

A mutated version of the *F. graminearum* xylanase gene (*mXyl*) encoding an inactive enzyme was constructed for transient expression in tobacco plants. The mutated xylanase lacked the enzymatic activity because a Glu (Glu214) in the catalytic site was substituted with a Ser (see Section 4). *Agrobacterium tumefaciens* strains harboring the *mXyl* expression vector or the pBI:GUS empty vector were infiltrated in tobacco leaves. RT-qPCR expression analysis carried out at 2, 3 and 4 days post infiltration showed an almost stable 6-fold increase of the *mXyl* transcript, compared to the tobacco actin used as housekeeping gene (Figure 2a). A radial gel diffusion assay performed with the proteins extracted from tobacco plants transiently expressing the mXyl confirmed the loss of its enzymatic activity (Figure 2b).

Four days after agro-infiltration, *P. syringae* pv. *tabaci* was inoculated on tobacco plants. Disease symptoms appeared as yellow-brown spots of variable size on the leaf surface. At four dpi, the symptomatic area of tobacco mXyl expressing leaves was significantly reduced by 48% (Figure 2c and Figure S1) compared to pBI:GUS control leaves.

After inoculation with *B. cinerea*, tobacco leaves transiently expressing the mXyl resulted as susceptible as the control leaves agro-infiltrated with the pBI:GUS plasmid (Figure 2d).

### 2.3. The Constitutive Expression of mXyl in Arabidopsis Transgenic Lines Provides Increased Resistance against *P. syringae* pv. *Maculicola* but Not against *B. cinerea*

**Figure 3:**
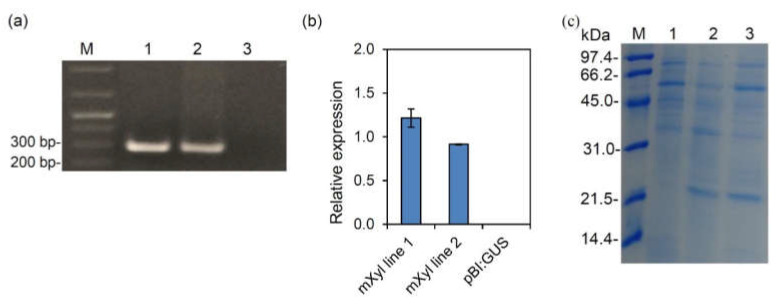
Characterization of Arabidopsis transgenic lines expressing mXyl: (**a**) PCR amplification performed using gene specific primers and genomic DNA of T0 Arabidopsis plants transformed with the mXyl construct. Amplicons were separated on 1% (*w/v*) agarose gel. Samples: M: marker; (1) mXyl line 1; (2) mXyl line 2; (3) negative control. (**b**) Relative expression level determined by qPCR of the *mXyl* gene in Arabidopsis transgenic lines transformed with *mXyl* or pBI:GUS. Each transcript was normalized with the Arabidopsis *ubiquitin* gene set to 1. Data represent the average ± mean standard error (SE, indicated by bars) of two qPCR experiments; (**c**) SDS-PAGE analysis of extracellular fluids of Arabidopsis plants expressing mXyl. Samples: M) Marker; (1) pBI:GUS; (2) mXyl line1; (3) mXyl line 2. A band of about 22 kDa corresponding to mXyl is visible in lanes 2 and 3.

The mutated xylanase was also expressed constitutively in transgenic Arabidopsis lines by transforming plants by the floral dip method with the Agrobacterium strain containing the *mXyl* encoding gene or the empty pBI:GUS vector. Seeds carrying the xylanase gene were selected by plating on Murashige and Skoog medium containing kanamycin. A total of two putative xylanase transgenic plants showed antibiotic resistance. A PCR performed using genomic DNA from the selected plants confirmed the successful transformation (Figure 3a).

Transgenic plants of the T2 generation showed no visible phenotypic differences compared to the control pBI:GUS plants (Figure S2). The RT-qPCR analysis confirmed the presence of the *mXyl* transcript in both transgenic lines (mXyl), with a peak of expression of about 1.2 folds for mXyl line 1 and 0.9 folds for mXyl line 2 compared to Arabidopsis *ubiquitin* used as a housekeeping gene (Figure 3b). Since the xylanase FGSG_03624 contains a predicted signal peptide of 19 residues, we verified the xylanase secretion in the extracellular fluid (EF) in the two mXyl lines. The glucose-6-phosphate dehydrogenase activity of the extracts was negligible, ruling out the cytoplasmic contamination of EF. When subjected to SDS-PAGE, the EF of both mXyl lines showed a 22 kDa band absent in control plants (Figure 3c). MALDI-TOF/TOF analysis confirmed the identity of the Xyl protein in the mXyl lines (protein accession: XYNB_GIBZE).

**Figure 4:**
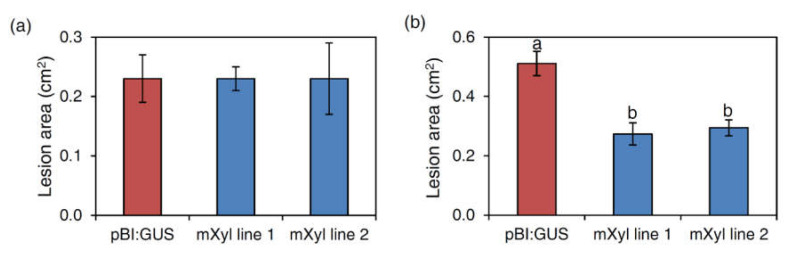
Infection experiments on Arabidopsis mXyl transgenic lines: (**a**) Lesion area (expressed in cm^2^) produced by *Botrytis cinerea* at 2 dpi. Bars indicate the standard error (SE) calculated from three independent infection experiments; (**b**) Lesion area produced by the bacterium *Pseudomonas syringae* pv. *maculicola* at 6 dpi. Lesion areas are expressed in cm^2^ ± standard error (SE) calculated from three independent infection experiments. All data were subjected to ANOVA analysis. When significant F values were observed (*p* < 0.05), a pairwise analysis was carried out by the Tukey honestly significant difference test. Letters correspond to the ranking of the Tukey test at *p* < 0.05.

Transgenic lines expressing mXyl were challenged with *B. cinerea* and *P. syringae* pv. *maculicola*. When inoculated with the fungus, no significant reduction of symptoms was observed compared to pBI:GUS plants (Figure 4a). Conversely, disease symptoms caused by *P. syringae* pv. *maculicola* significantly decreased by 46.5% and 42.5% in mXyl line 1 and mXyl line 2, respectively, compared to pBI:GUS control plants (Figure 4b and Figure S3).

### 2.4. The Xyl Protein Does Not Affect P. syringae pv. Maculicola Growth

The efficacy of the FGSG_03624 in reducing *P. syringae* pv. *maculicola* symptoms in transgenic Arabidopsis plants prompted us to investigate if this effect could result from a direct toxic effect of the xylanase against the bacterium. For this purpose, we measured the growth of *P. syringae* pv. *maculicola* in the presence of Xyl using an in vitro assay with the vital dye resazurin, a colorimetric indicator of bacterial cell viability. At all the time points analyzed (24, 48 and 72 h of co-incubation), the xylanase did not interfere with the growth of the pathogen (Figure S4).

### 2.5. SA-Dependent Defense Pathway Is Strengthened in Arabidopsis mXyl Lines after *P. syringae* Inoculation

**Figure 5:**
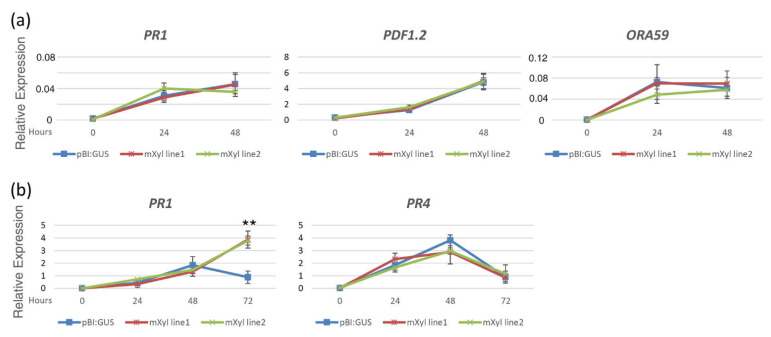
Pathogen-triggered gene induction in Arabidopsis mXyl or pBI:GUS transgenic lines. The expression of selected defense-related marker genes (*PR1*, *PR4*, *PDF1.2*, *ORA59*) was analyzed by qPCR at the indicated time points in control plants (pBI:GUS) and mXyl transgenic lines 1 and 2 upon infection with *Botrytis cinerea* (**a**) or *Pseudomonas syringae* pv. *maculicola* (**b**). Transcript levels are shown as the mean of three independent experiments [± standard deviation (SD); n = 4 in each experiment] normalized to UBQ5 expression. Asterisks indicate statistically significant differences between control and mXyl lines according to Student’s t-test (** *p* < 0.05).

Having excluded a direct toxic effect of the xylanase against *P. syringae* pv. *maculicola*, we first investigated whether the constitutive expression of mXyl in Arabidopsis could determine an alteration of plant defense responses. Complex interactions between hormonal signaling pathways coordinate pathogen-induced immune responses in plants. Therefore, the expression of defense-related genes was analyzed in pBI:GUS and mXyl lines challenged with *B. cinerea* or with *P. syringae* pv. *maculicola*. In particular, we analyzed the expression of (i) *OCTADECANOID-RESPONSIVE ARABIDOPSIS AP2/ERF 59* (*ORA59*), encoding an ET/JA-regulated transcription factor required for both the expression of *PLANT DEFENSIN 1.2* (*PDF 1.2*) and basal resistance to *B. cinerea* [25,26], (ii) *PR4*, an ET/JA-dependent signaling pathway marker, (iii) *PR1*, a marker for the SA-dependent immune responses. Basal expression of these genes was not affected by the *mXyl* constitutive expression (Figure 5; time point 0). Leaves of adult mXyl and control plants were excised and drop-inoculated with *B. cinerea* conidia. Transcript levels of *ORA59*, *PDF1.2* and *PR1* were similarly affected in mXyl transgenic lines and control plants both at 24 and 48 h post-inoculation (hpi; Figure 5a). After 48 h, the gene expression analysis was not carried out due to the complete maceration of the leaf tissue caused by the fungal infection.

Transcript analyses were also performed on 4-week-old leaves of pBI:GUS and mXyl lines challenged with *P. syringae*. Among the considered genes, the expression of *ORA59* and *PDF1.2* was not significantly altered by the pathogen with respect to mock-infiltrated tissues in both mXyl and control transgenic lines (not shown), whereas *PR4* was induced after pathogen inoculation without significant differences among the mXyl and control lines. The only difference in the expression pattern was identified for the *PR1* gene. *Pseudomonas syringae* highly induced its expression in both control and transgenic leaves at 24 and 48 hpi; interestingly, at 72 hpi, the expression of *PR1* increased in the mXyl lines, whereas it significantly diminished in the control leaves (Figure 5b).

We also verified whether well-known microbe-associated molecular patterns (MAMPs) or damage-associated molecular patterns (DAMPs) induce defense responses in mXyl lines. To this aim, we used flg22, derived from the bacterial flagellin, as a MAMP, or OG fragments of about 10–16 residues of α-1,4-D-galactopyranosyluronic acid residues as a DAMP. Seedlings were examined for the expression of genes that are induced early in response to flg22 or OGs [27]. We chose *RET-OX*, which encodes a protein with homology to reticuline oxidases [28], the flagellin-responsive receptor-like kinase gene *FRK1* and *CYP81F2*, encoding a cytochrome P450 involved in indol-3-yl-methyl glucosinolate catabolism [29]. Expression of these genes was examined in Arabidopsis mXyl lines as well as in pBI:GUS plants. In response to both elicitors, transcript accumulation of the three genes examined was not significantly different between the transgenic and control pBI:GUS plants (Figure S5).

MPK3 and MPK6 are also activated in response to OGs or flg22 [30], and MPK6 is also associated with resistance to *B. cinerea* [31]. We, therefore, investigated whether the activation of MPKs changes in plants expressing mXyl after OGs or flg22 treatments. As shown in Figure S5, no significant differences were detected among mXyl and control lines.

### 2.6. Spray Treatment of Wheat Spikes with Xyl Confers Increased Resistance to FHB and Decreases Fungal Biomass in Kernels

**Figure 6:**
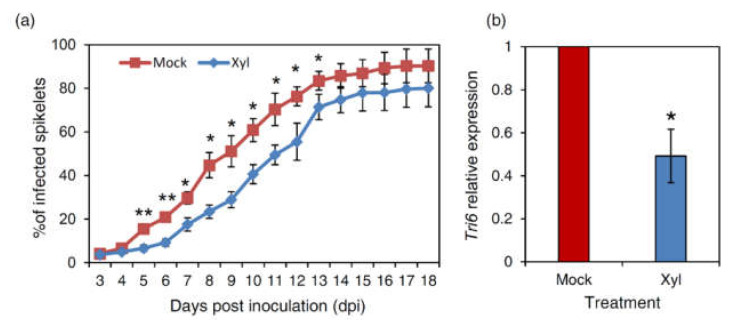
Inoculation experiments with *Fusarium graminearum* on durum wheat plants cv. Svevo exogenously treated with the purified FGSG_03624 xylanase (Xyl) or with water (mock). (**a**) Time-course analysis of Fusarium Head Blight symptoms, expressed as a percentage of infected spikelets, and (**b**) quantification of fungal biomass in semolina. Relative quantification of *F. graminearum* DNA in wheat semolina was based on *Tri-6* gene quantification by qPCR experiments. Wheat *actin* was used as a housekeeping gene. Data represent the average ± standard errors of three independent experiments. Asterisks indicate significant differences according to Student’s *t*-test at *p* ≤ 0.01 (**) or *p* ≤ 0.05 (*).

Spikes of durum wheat were treated with the purified Xyl or with water as a negative control and, after two days, the spikes were point-inoculated with *F. graminearum*. Disease progression was different between the two groups of plants. At 4–6 days from fungal inoculation, several spikes treated with Xyl showed symptoms restricted at the inoculation site, while in the control plants, symptoms were more severe, affecting more spikelets (Figure S6). Subsequently, symptoms also began to extend to the nearby spikelets in the xylanase treated plants. Compared to the control, the delay in disease progression in the xylanase treated spikes was maximum at 9 dpi, when the severity reduction was about 43% (Figure 6a). The significant difference in symptoms severity was maintained from 5 dpi up to 11 dpi, while from 13 dpi to the end of experiments (18 dpi), the difference was no longer significant (Figure 6a).

At 18 dpi, the fungal biomass was quantified by quantitative PCR using specific primers for the fungal *Tri-6* gene and the total DNA extracted from the whole semolina of the Xyl treated and control spikes. Xyl-treated spikes exhibited 50% reduced fungal biomass compared to control (Figure 6b). Thus, the delayed progression of FHB symptoms in xylanase-treated plants appears associated with a reduced accumulation of fungal biomass in the caryopses.

### 2.7. Callose Deposition in Response to *F. graminearum* Infection Highlight a Priming Effect of Xyl

**Figure 7:**
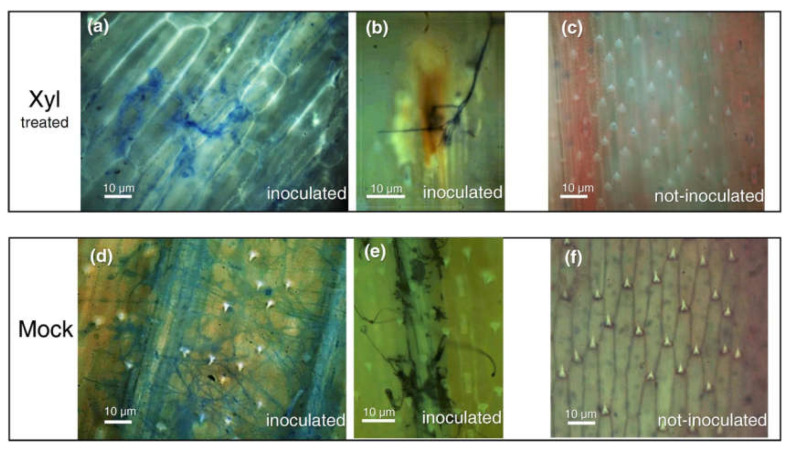
Callose deposition microscopically visualized via aniline blue staining in *Triticum durum* cv. Svevo lemmas obtained from spikes sprayed at the pre-anthesis stage with FGSG_03624 (Xyl) or water (mock) as negative control and, afterward, point-inoculated at anthesis stage with *Fusarium graminearum* conidia. Fluorescence by callose deposition is evident in (**a**,**b**). Fungal hyphae are also blue-stained by aniline (**a**,**b**,**d**,**e**). Not inoculated lemmas were also observed by microscope (**c**,**f**). Aniline blue staining and callose visualization were performed four days after fungal inoculation.

To investigate the mechanism underlying the ability of the Xyl to delay the disease progression and reduce *F. graminearum* biomass, we investigated the accumulation of callose, one of the first defense mechanisms activated by plants to restrict the pathogen growth. Callose deposition was determined by aniline blue and UV epifluorescence microscopy analyses of lemmas of durum wheat spikes treated with Xyl or water (mock) and inoculated or not with *F. graminearum*. Four days after inoculation with *F. graminearum*, callose deposition was particularly intense in lemma tissues of the Xyl-treated spikes (Figure 7a), showing many penetration attempts by the pathogen (Figure 7b). In contrast, callose was weakly detectable in lemmas of mock-treated spikes (Figure 7d,e). In spikes not inoculated with the fungus, callose deposition was not detected both in Xyl and mock-treated spikes (Figure 7c,f).

## 3. Discussion

The present paper aims to establish whether the FGSG_03624 xylanase (Xyl) may be exploited to protect plants from diseases. The issue has been addressed with various experimental systems. Initially through the exogenous application of the xylanase protein in the model plant Arabidopsis, then by the transient expression of the xylanase in tobacco, and finally through its constitutive expression in Arabidopsis. In all these experiments, the plants were challenged with the necrotrophic fungus *B. cinerea* and the hemibiotrophic bacterium *P. syringae*. Later, to deal with a disease of greater economic interest, we evaluated the effect of administering the xylanase to durum wheat spike to contain FHB, one of the most important diseases of this crop. In Arabidopsis and tobacco expression experiments, we used an inactive version of the xylanase to avoid undesirable degradation effects on the plant cell wall. Indeed, the *F. graminearum* Xyl induces necrosis and hydrogen peroxide accumulation in wheat [18] independently from its catalytic activity, as reported for other fungal xylanases [15,16,32].

The main experimental result was that Arabidopsis and tobacco plants reacted similarly to the two pathogens whatever the mode of xylanase administration: compared to the controls, the symptomatic disease area was significantly reduced after inoculation with *P. syringae* and did not change after inoculation with *B. cinerea*.

We excluded the sensitivity of the bacterium to Xyl as the cause of the reduced severity in the Xyl plants since we verified that Xyl does not impair the *P. syringae* growth.

The hypothesis of the activation of defense responses effective against *P. syringae* and ineffective against *B. cinerea* was addressed in Arabidopsis plants constitutively expressing the xylanase gene (mXyl lines). Even though they contain the xylanase protein in the apoplast, these plants exhibit a phenotype similar to that of the control pBI:GUS plants. The defense genes analyzed were selected among those regulated by the SA (*PR1* gene) or ET/JA (*PR4*, *ORA59*, and *PDF1.2*) hormonal pathways. Before the pathogens inoculation, the mXyl lines did not significantly increase the expression level of the selected defense genes compared to the control pBI:GUS plants. When challenged with *P. syringae*, both control and mXyl lines displayed a much higher expression of the *PR1* gene, and the induction of the *PR1* gene was significantly higher at 72 hpi in both mXyl lines compared to the control plants. In contrast, the ET/JA responsive genes were not induced during *P. syringae* infection or, as observed with *PR4*, were similarly regulated in mXyl lines and control plants.

*PR1* is a well-known marker of SA-dependent immune response that could be associated with the increased resistance observed against the hemibiotrophic bacterial pathogen *P. syringae* [33]. Similarly, induction of SA-regulated pathogenesis-related genes, namely *PR1* and *PR5*, was recorded in tobacco plants infiltrated with the BcXyn11 xylanase [15], a protein structurally similar to Xyl [18,34]. Thus, regardless of the different experimental approaches in supplying the fungal xylanase, results seem consistent with the activation of an SA-regulated immune response capable of contrasting the infection by *P. syringae*. However, while the exogenous application of *Bc*Xyn11 to tobacco activates the PR responses few h after the infiltration [15], the constitutive expression of the FGSG_03624 xylanase in Arabidopsis did not induce *PR1* expression. This SA-defense PR marker was boosted only after the pathogen inoculation. A similar mechanism of defense activation only after pathogen infection has been reported in transgenic plants expressing some regulation factors [35,36].

To evaluate whether the perception system of molecular signals released during plant-pathogen interaction is more reactive in the mXyl lines than in the control plants, we treated the transgenic Arabidopsis plants with the well-known elicitors OGs and flg22. The first is a mixture of oligomers released by the degradation of the plant cell wall pectins; the second is the active epitope of flagellin, a PAMP of gram-negative bacteria. Results showed that the perception system triggered both by OGs or flg22—determined through measures of phosphorylation of MPK3 and MPK6 and expression of some immune marker gene—is active and not altered in Arabidopsis mXyl lines constitutively expressing the xylanase in comparison to control plants. Therefore, the presence of FGSG_03624 in Arabidopsis does not alter the pathogen detection nor determines a faster activation of the immune signal transduction. In conclusion, these experiments did not provide any decisive element for interpreting the different responses of the mXyl lines to *B. cinerea* and *P. syringae* infection, and the cause of the protracted expression of *PR1* in the mXyl lines inoculated with *P. syringae* remains an open question.

In the plants inoculated with *B. cinerea*, the expression of *PR1* was low, and no differential expression of this gene was observed compared to the control. This result is not surprising, considering that *PR1* does not play a role against necrotrophic pathogens [37,38,39]. The expression of the transcription factor *ORA59* and the plant defensin *PDF1.2*, two genes regulated by the ET/JA pathway involved in the basal defense against necrotrophic pathogens, agrees with the lifestyle of *B. cinerea*. The observation that these genes appeared similarly expressed in the mXyl lines compared to the control plants also agrees with the similar disease severity measured after *B. cinerea* inoculation. Notwithstanding these defense genes are induced after *B. cinerea* inoculation, their expression level may be too low to counteract the development of symptoms by this fast-growing pathogen, which is known to secrete an array of virulence and necrosis factors to overcome plant defense responses [40].

The exogenous treatment with Xyl on durum wheat spikes 48 h before inoculation with *F. graminearum* successfully reduced FHB symptoms only at the early stages of wheat spike infection. At the end of the experiment, the difference compared with mock-treated plants was no longer significant, but a significantly reduced fungal biomass highlights the capacity of the spike tissue to hamper the progression of fungal infection. Our experiments also showed a deposition of callose primed by the exogenous treatment of wheat spikes with Xyl. Several evidences report that callose deposition can be primed in elicitor-treated plants [41]. Besides, thick-layered callose apposition and formation of papillae were found to be higher in the FHB resistant “Sumai3” genotype compared to susceptible cultivars [42,43]. Therefore, the mechanism involved in delaying the fungus spreading in the Xyl treated spike tissue could be related to a rapid callose deposition.

Interestingly, the callose deposition observed in Xyl treated wheat spikes occurs only after *F. graminearum* infection, evoking what we detected in the Arabidopsis mXyl plants, where a sustained *PR1* gene expression occurs only after *P. syringae* infection. This result appears to be related to the trophic lifestyle of the pathogen. Indeed, similarly to *P. syringae*, *F. graminearum* is considered a hemibiotrophic pathogen [44].

In conclusion, our work demonstrates that the FGSG_03624 xylanase can improve plant resistance responses depending on the type of pathogen considered. The xylanase strengthened the immune response in Arabidopsis against *P. syringae* by extending the activation of the SA-dependent defense pathway(s) and stimulated callose deposition in wheat against *F. graminearum*. Remarkably, in both cases, defense mechanisms were activated only after the pathogen challenge. This priming effect is interesting in view of using Xyl for crop protection without incurring in fitness cost for the plant. In this regard, it has been recently shown that short epitopes on fungal xylanase are the likely candidates for inducing the defense responses in plants [15]. These findings may provide the chemical basis for the development of new peptides exploitable in crop protection.

## 4. Materials and Methods

### 4.1. Xylanase Treatment of Arabidopsis Leaves

Leaves of *A. thaliana* plants (8–12 leaves stage) were treated by spraying abaxial leaf surfaces with the *F. graminearum* xylanase FGSG_03624 (100 μg mL^−1^) or water as control, both supplemented with pinolene 0.04% (*v/v*). The heterologously expressed FGSG_03624 xylanase used in the experiments was produced and purified as described in [18].

For treatments of seedlings with OGs or flg22, seeds were surface sterilized and germinated in multiwell plates (approximately 10 seeds per well) containing 2 mL per well of Murashige and Skoog (MS) medium (Sigma-Aldrich, St. Louis, MO, USA) [45] supplemented with 0.5% sucrose. After 9 days, the medium was adjusted to a final volume of 1 mL and treatments with water, OGs (100 µg/mL) or flg22 (10 nM) were performed after 24 h.

### 4.2. Production of the mXyl Construct and Agrobacterium Transformation

The *F. graminearum FGSG_03624* gene encoding an endo-1,4-beta-xylanase of 228-amino acids (Figure S7a) was first amplified using cDNA from wheat spikes infected by *F. graminearum* by using the primers 03624Fc and 03624Rc (Table S1) and repeating for 35 times the following cycle: 30 s at 94 °C, 30 s at 55 °C, 1 min at 72 °C. The DNA fragment was purified and cloned into pGEM-T Easy Vector (Promega, Madison, WI, USA). Since the catalytic activity of the protein is due to two Glu residues (conserved in all the xylanases of the GH11 family) at positions 122 and 214, to obtain the FGSG_03624 protein without enzymatic activity, a glutamic acid codon (GAG) coding for the Glu (E) residue at position 214 was changed to serine (S) codon (TCG) (Figure S7b). The site-directed mutagenesis was performed by PCR amplification with primer pair XYL-F XbaI and XYL-R SacI (Table S1) containing a mutation in the reverse primer. PCR conditions were 30 sec at 94 °C and 2 min at 72 °C (annealing and extension steps) for 35 cycles. The mutated gene (*mXyl*) was then digested with SacI and XbaI and ligated into the pBI121expression vector cut with the same restriction enzymes. The expression cassette was put under the control of the constitutive *CaMV 35S* promoter and *NOS* terminator. The *NPTII* gene was used for selection with kanamycin. Empty pBI:GUS vector used as a negative control was from Takara bio (Kusatsu, Japan). Transformation of *Agrobacterium tumefaciens* (strain GV3101, resistant to rifampicin and gentamicin) was performed as reported in [46] and described by [47].

### 4.3. Tobacco and Arabidopsis Transformation, Selection of Arabidopsis Transgenic Plants, and Characterization of Tobacco and Arabidopsis Transformants

Agrobacterium-mediated transformation of *Nicotiana tabacum* (ecotype SR1) and *Arabidopsis thaliana* (ecotype Col-0) were performed by agro-infiltration and floral dip methods. Agro-infiltration of *N. tabacum* leaves with *A. tumefaciens* strain harboring the *mXyl* gene or the pBI:GUS vector as control was performed as reported in [45]. Leaves were infiltrated with the *A. tumefaciens* strain harboring *mXyl* or pBI:GUS. The Infiltration area outlined with a black marker were collected after 24, 48, 72, and 96 h from agro-infiltration for expression analysis.

The genomic DNAs of the kanamycin-resistant transformed Arabidopsis plants were extracted by using the “DNeasy Plant MiniKit” (Qiagen, Hilden, Germany) according to the manufacturer’s instructions. The presence of the mutated *mXyl* in Arabidopsis transgenic plants (mXyl) was evaluated by PCR using the primer pair 03624RTfor/03624RTrev (Table S1). The PCR reaction, performed in a 25 μL volume, consisted of 3 min at 94 °C, followed by 35 cycles of 94 °C for 30 s, 54 °C for 30 s and 72 °C for 3 min.

### 4.4. Plant, Fungal, and Bacterial Growth Conditions, Infection Assays and Fungal Biomass Quantification

*Triticum durum* cv. Svevo, *A. thaliana*, and *N. tabacum* plants were grown in a controlled environment at 20–22 °C with a 16 h photoperiod. The bacterial pathogens *Pseudomonas syringae* pv. *maculicola* and *P. syringae* pv. *tabaci* were grown in King’s B medium [48] at 200 rpm and 28 °C. The fungal pathogen *Fusarium graminearum* (strain 3824) was cultured on synthetic nutrient agar (SNA) medium at 25 °C [49] to produce macroconidia. *Botrytis cinerea* (strain B05.10) was grown on potato dextrose agar (PDA; Difco Laboratories, Detroit, MI, USA) at 28 °C.

Inoculation experiments of exogenously treated or transgenic Arabidopsis leaves with *P. syringae* pv. *maculicola* or *B. cinerea* were performed as reported in [46]. Inoculation was performed four days after exogenous treatment with Xyl. Disease symptoms caused by *P. syringae* pv. *maculicola* or *B. cinerea* were evaluated at 6 dpi and 2 dpi, respectively, as reported in [46].

Inoculation experiments of *mXyl* agro-infiltrated tobacco leaves with *P. syringae* pv. *tabaci* or *B. cinerea* were performed four days after agro-infiltration as reported in [46]. Disease symptoms were analyzed at 4 dpi for *P. syringae* pv. *tabaci* and at 3 dpi for *B. cinerea* as reported in [46].

Inoculation experiments with *F. graminearum* were performed by single-spikelet inoculation two days after exogenous treatment of the wheat heads with a solution containing 100 μg mL^−1^ of Xyl or water as a negative control supplemented with pinolene 0.04% (*v/v*). Inoculation experiments and disease symptom analyses were carried out as described by [50]. Quantification of fungal biomass in infected caryopses was performed via qPCR by analyzing the *Tri-6* gene [51] as reported by [8]. At the end of each of the three infection experiments (18 dpi), all dry caryopses of Xyl and mock treated plants were pooled to make whole wheat semolina, and total DNA from whole flour was extracted for qPCR. Wheat *actin* (accession number AB181991) was used as the housekeeping gene.

In each plant inoculation experiment, at least 12 plants for each treatment or genotype were used.

### 4.5. Bacterial Growth Inhibition Assay

To verify the sensitivity of *P. syringae* pv. *maculicola* to FGSG_03624, an in vitro growth assay with the vital dye resazurin was carried out as reported in [52]. Reactions were performed in a 200 µL volume containing bacterial cells (OD_600_ 0.2) diluted in King’s B medium and 100 ng of the purified FGSG_03624. Bacterial growth was expressed as the net absorbance values of samples with *P. syringae* pv. *maculicola* alone or co-incubated with FGSG_03624 calculated with respect to the absorbance of the control at 0 h and changed in sign. Two independent experiments were performed with three biological replicas.

### 4.6. Gene Expression Analyses

RNA was extracted from 100 mg of transgenic Arabidopsis leaves or infiltrated tobacco leaves by using the RNeasy Plant Mini Kit (Qiagen, Hilden, Germany) following the manufacturer’s instructions. RNA was treated with RQ1 DNaseI (Promega, Madison, USA) following the manufacturer’s instructions and quantified both by spectrophotometer and a denaturing gel. First-strand cDNA was synthesized using ImProm-II reverse transcriptase (Promega, Madison, WI, USA) according to the manufacturer’s instructions by mixing 500 ng of an oligo-dT (15/18 thymine) reverse primer with 0.5 μg target RNA.

The expression of mXyl in Arabidopsis transgenic lines and tobacco agro-infiltrated plants was performed by RT-qPCR (Rotor-Gene Q 2plex, Qiagen, Hilden, Germany) using the primer pair 03624RTfor/03624RTrev (Table S1) as reported in [46]. Each transcript was normalized with the Arabidopsis *ubiquitin* gene (AY139810.1) amplified with primer pair UBQ5-F/UBQ5-R (Table S1) or with the tobacco *actin* gene (U60495.1) amplified with primer pair Tob103-For/Actin Tob103-Rev (Table S1). Relative expression was analyzed as reported in [46].

For the analyses of immune-related gene markers, 10-d-old seedlings, treated with OGs (100 µg/mL) or flg22 (10 nm) for 30, 60 or 180 min, or leaf tissues, infected with *B. cinerea* for 24 or 48 h or with *P. syringae* pv. *maculicola* for 24, 48 and 72 h, were frozen in liquid nitrogen and homogenized with a MM301 Ball Mill (Retsch GmbH, Haan, Germany). Total RNA was extracted from at least three independent replicates, each composed by 20 seedlings or at least three adult leaves from different plants, with TRIzol Reagent (Thermo Fisher Scientific, Waltham, MA, USA) according to the manufacturer’s protocol. DNase treatment was performed as reported above. According to the manufacturer’s instructions, cDNA was synthesized using iScript reverse transcriptase (Biorad, Hercules, CA, USA) and amplified from 50 ng of total RNA in a 30-µL reaction mixture containing 1× SsoAdvanced Universal SYBR green Supermix (Biorad, Hercules, CA, USA) and 0.5 µM of each primer. qRT-PCR analysis was performed by using a CFX96 Real-Time System (Biorad, Hercules, CA, USA). Three technical replicates were performed for each sample, and data analysis was done using LinRegPCR software. The expression levels of each gene, relative to *UBIQUITIN5*, were determined using a modification of the Pfaffl method [53] and expressed in arbitrary units. Primer sequences are shown in Table S1.

### 4.7. Extracellular Fluids Extraction and Analysis

Extracellular fluids (EFs) were collected from Arabidopsis 6-week-old rosette leaves as described previously [54] and according to [55]. Briefly, Leaves were stacked in the bottom of a 10-mL plastic syringe and washed with McIlvaine’s buffer at pH 5.0 (0.2 M disodium hydrogen phosphate and 0.1 M citric acid) for 5 min and then vacuum-infiltrated for 10 min with the same buffer. EF was recovered by centrifuging the vacuum-infiltrated leaves at 500× *g* for 5 min at 4 °C. Contamination of EFs by cytoplasmic components was ruled out by measuring glucose-6-phosphate dehydrogenase activity, according to [56]. Afterward, the infiltrated buffer was subjected to PD10 desalting process against 50 mM Ammonium acetate pH 7.0 used for SDS-PAGE analysis. MALDI-TOF/TOF analysis for protein identification was performed as reported in [57].

### 4.8. MAPK Activation Assay

Control and transgenic seedlings were germinated and grown for 10 days on 1⁄2 MS medium supplemented or not with 0.5% saccharose. After treatment with OGs (100 µg/mL) or flg22 (10 nm) for 5, 15 or 30 min, proteins were extracted with 50 mM Tris, pH 7.5 200 mM NaCl, 1 mM EDTA, 10% (*v/v*) glycerol, 0.1% (*v/v*) Tween 20, 1 mM PMSF, 1 mM dithiothreitol, 1× phosphatase inhibitor mixture 2 (Sigma-Aldrich, St. Louis, MO, USA) and 1× protease inhibitor mixture (Sigma-Aldrich, St. Louis, MO, USA). Proteins (30 μg) were resolved on 7.5% polyacrylamide gels and transferred onto a nitrocellulose membrane (Biorad, Hercules, CA, USA). Primary antibody against phospho- p44/42 MAP kinase (1:2500; Cell Signaling Technology, Danvers, MA, USA) were used with HRP-conjugated anti-rabbit as a secondary antibody (1:8000; GE Healthcare, Buckinghamshire, UK).

### 4.9. Callose Deposition Analysis

Callose deposition analysis was carried out on lemmas from wheat spikes treated with 100 μg mL^−1^ of purified FGSG_03624 or water as negative control and inoculated with *F. graminearum* as described above. Plants treated with Xyl or water but not inoculated were included in the experiment as a control. Spikes were dissected four days after inoculation, and lemmas were fixed and de-stained overnight in 1:3 acetic acid/ethanol. De-stained lemmas were incubated overnight in 0.01% aniline blue dissolved in 150 mM K_2_HPO_4_. Callose deposition was evaluated by fluorescence microscopy using an exciter filter of 330–385 nm and a barrier filter of 400 nm (Olympus BX50, Tokyo, Japan). Three biological and three technical replicas were performed.

### 4.10. Statistical Analysis

Data were analyzed with Student’s *t*-test or ANOVA by using the SYSTAT12 software (Systat Software Incorporated, San Jose, CA, USA). When significant F values were observed (*p* < 0.05), a pairwise analysis was carried out by the Tukey Honestly Significant Difference test (Tukey test).

## Data Availability

The data that support the findings of this study are available from the corresponding author upon reasonable request.

## Supplementary Materials

The following are available online at https://www.mdpi.com/article/10.3390/ijms221910811/s1.
