# Supplementary material for: The Fusarium graminearum FGSG_03624 Xylanase Enhances Plant Immunity and Increases Resistance against Bacterial and Fungal Pathogens
Source: Int J Mol Sci. 2021 Oct 6;22(19):10811. doi: 10.3390/ijms221910811 (PMC8509205; doi:10.3390/ijms221910811)
Supplement: Supplementary file 1 [file ijms-22-10811-s001.zip › ijms-1391006-supplementary.pdf]

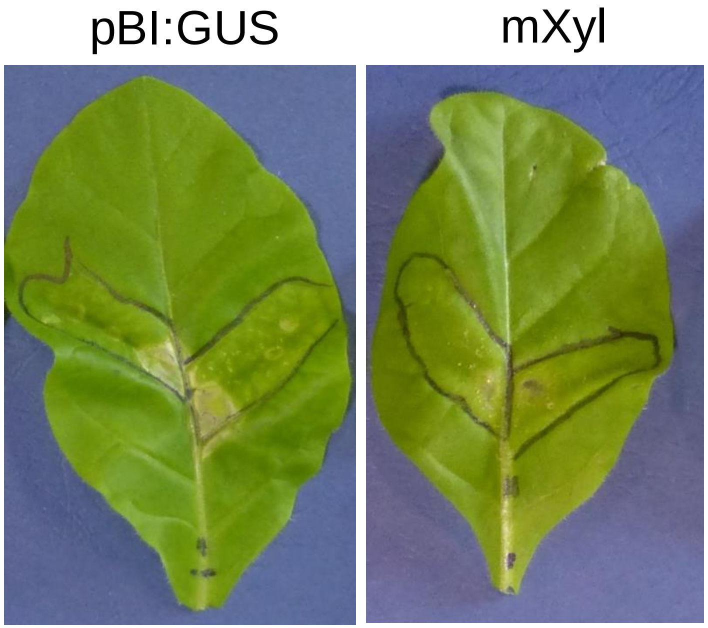

Figure S1. Lesion area produced by *Pseudomonas syringae* pv. *tabaci* on *Nicotiana tabacum* cv. SR1 leaves agro-infiltrated to express the mutated xylanase FGSG_03624 (mXyl) or with pBI:GUS as control at 4 dpi.

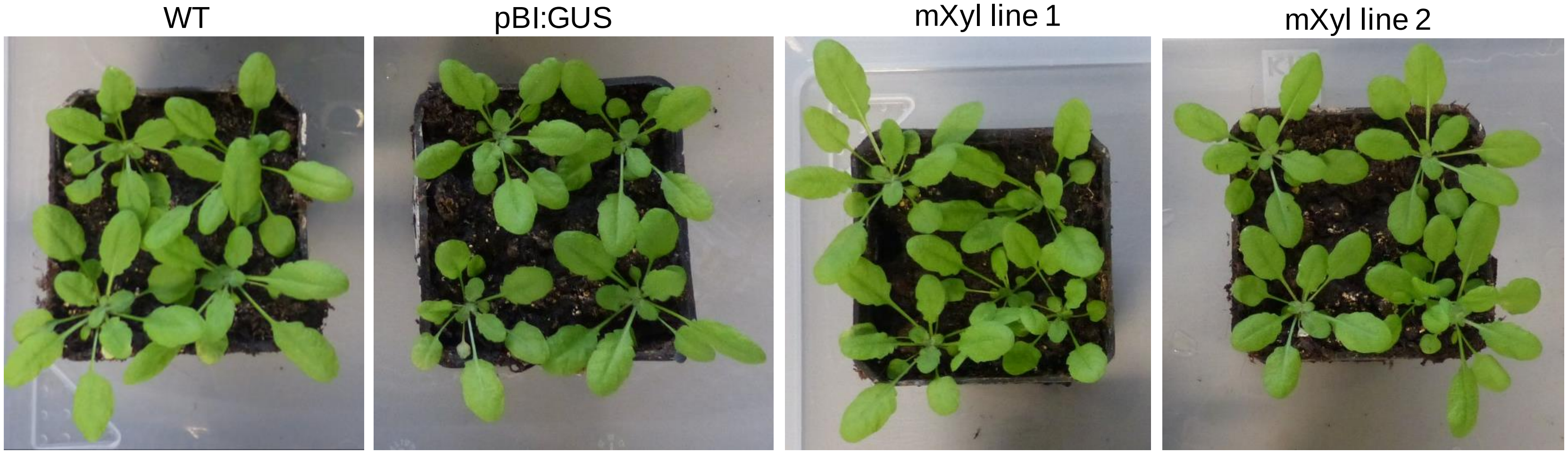

Figure S2. Phenotypic analysis of Arabidopsis transgenic lines expressing the enzymatically inactive FGSG_03624 xylanase (mXyl line 1 and mXyl line 2) or pBI:GUS (empty vector) as compared to Col-0 plants (WT).

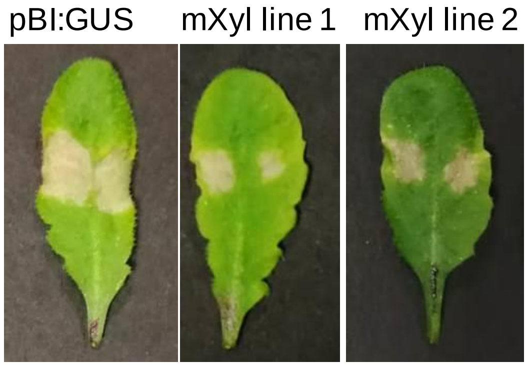

Figure S3. Lesion area produced by the bacterium *Pseudomonas syringae* pv. *maculicola* on Arabidopsis transgenic lines expressing the enzymatically inactive FGSG_03624 xylanase (mXyl line 1 and mXyl line 2) or pBI:GUS (empty vector) at 6 dpi.

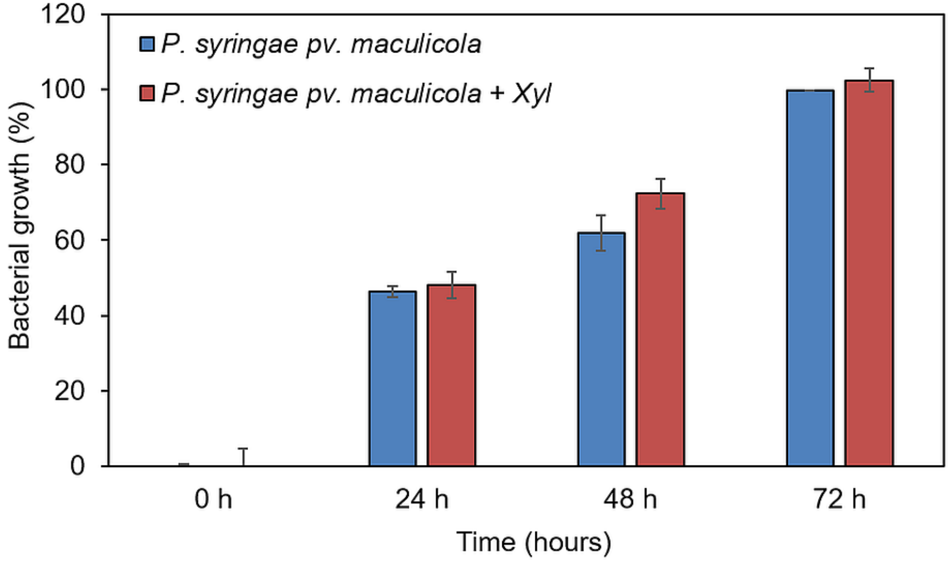

Figure S4. In vitro growth assay to test possible direct toxicity of FGSG_03624 (Xyl) against *Pseudomonas syringae* pv. *maculicola*. Bacterial growth was expressed as the net absorbance values of samples with *P. syringae* pv. *maculicola* alone or co-incubated with FGSG_03624 calculated with respect to the absorbance of the control at 0 h and changed in sign. Data represent the average ± standard error of two independent experiments with three technical replicates.

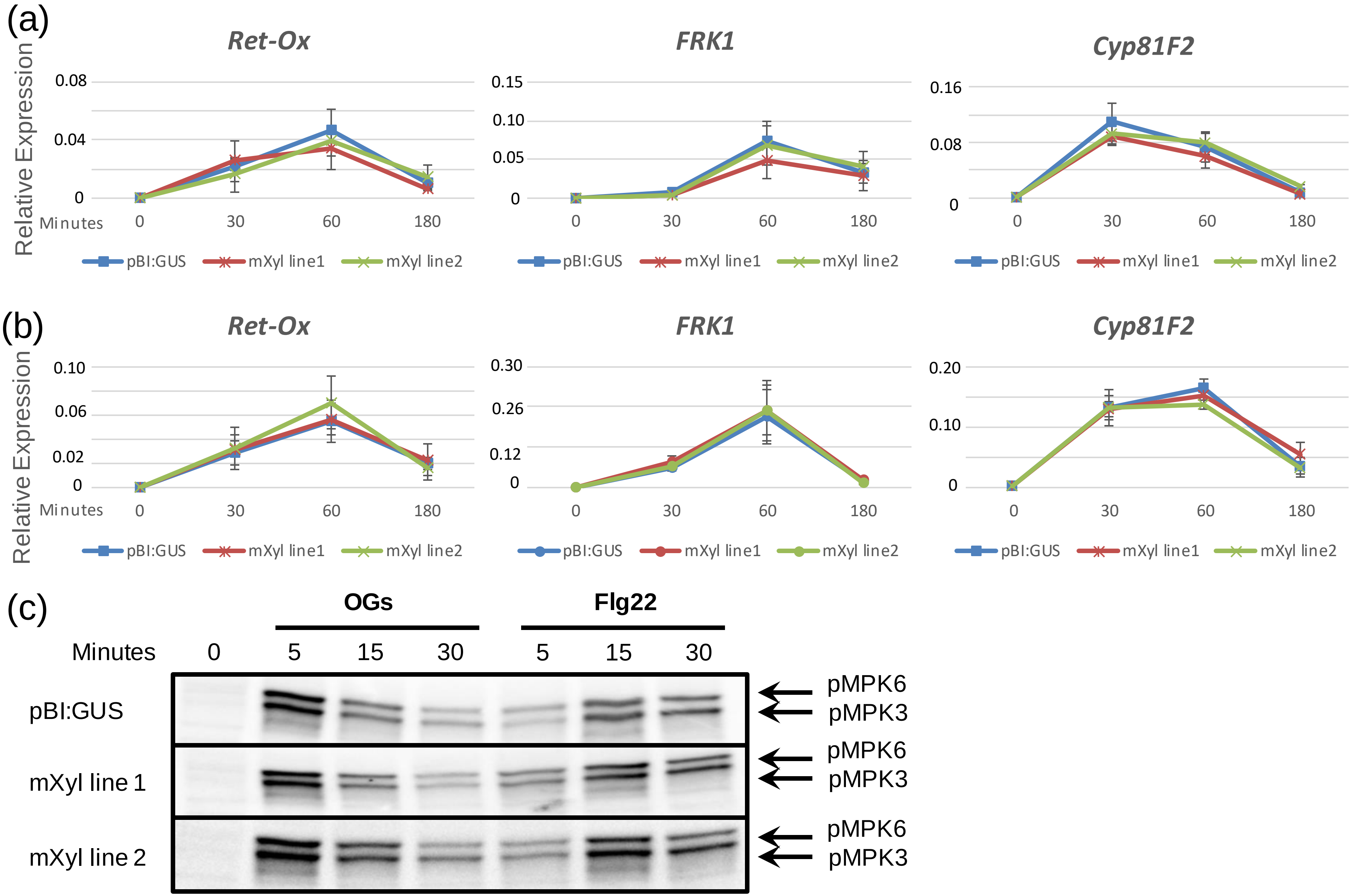

Figure S5. Elicitor-triggered gene induction (a, b) and MPK activation (c) are not affected by mXyl expression in transgenic Arabidopsis plants. The expression of defense-related marker genes was analyzed by qPCR in control plants (pBI:GUS) and mXyl transgenic lines 1 and 2 upon treatment with (a) oligogalacturonides (OGs) or (b) flg22. (c) Levels of phosphorylated MPK3 and MPK6 (pMPK3 and pMPK6) after elicitation with OGs or flg22 at the indicated time points were determined by immunoblot analysis using an anti-p44/42-ERK antibody. An equal amount of proteins was used for each sample. The experiments were repeated three times with similar results.

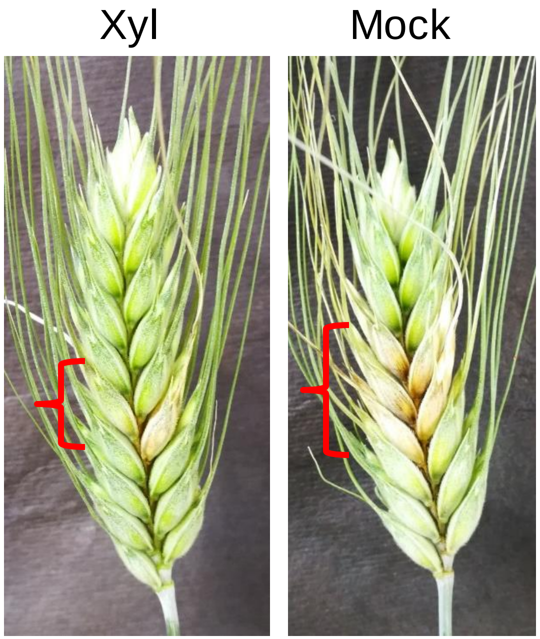

Figure S6. Representative Fusarium Head Blight symptoms in *Fusarium graminearum* inoculated wheat spikes at 6 dpi following exogenous treatment with the FGSG_03624 xylanase (Xyl) or with water (mock).

(a)

```
1   MVSFTYLLAA VSAVTGAVAA PNPTKVDAQP PSGLLEKRTS PTTGVNNGFY FSFWTDTPSA
61  VTYTNGNGGQ FSMNWNGNRG NHVGGKGWNP GAARTIKYSG DYRPNGNSYL AVYGWTRNPL
121 VEYYIVENFG TYNPSSGAQK KGEINIDGSI YDIAVSTRNC APSIEGDCKT FQQYWSVRRN
181 KRSSGSVNTG AHFNAWAQAG LRLGSHDYQI LAVEGYQSSG QATMTVSG
```

(b)

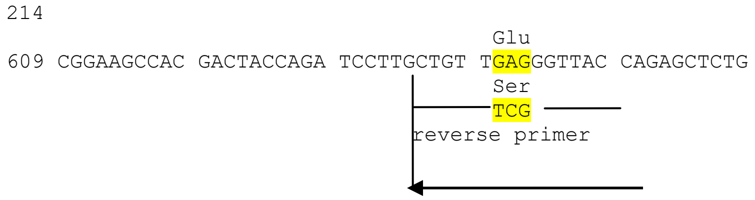

Figure S7. Amino acid sequence of the *Fusarium graminearum* xylanase FGSG_03624 and site-directed mutagenesis of the codon involved in the catalytic activity. (a) The first 19 underlined amino acids represent the signal peptide; the two highlighted Glu (E) residues are essential for enzymatic activity. (b) Using a mutated reverse primer represented by the arrow, site-directed mutagenesis performed by PCR to change the Glu codon at position 214 to Ser codon.

Table S1 List of primers used in this work.

PRIMER NAME	PRIMER SEQUENCE (5'-3')
**Primers for cloning *FGSG_03624* gene**	
03624Fc	ATGGTCTCCTTCACCTACCT
03624Rc	TATCCAGAGACAGTCATGGT
XYL-F XbaI	AATAATCTAGAATGGTCTCCTTCACCTACCTTCTCGCCGCTGTCTCGGCC
XYL-R SacI	AATAAGAGCTCTTATCCAGAGACAGTCATGGTAGCCTGGCCACTGCTCTGGTAACCCGAAAC
03624RTfor	GTCTCCTTCACCTACCTTCTC
03624RTrev	TCCATCCCTTACCACCGA
UBQ5-F	GTGGTGCTAAGAAGAGGAAGA
UBQ5-R	TCAAGCTTCAACTCCTTCTTT
Actin Tob103-For	TAGGCTGGATTTGCTGGTGA
Actin Tob103-Rev	TCCATGTCATCCCAGTTGCT
RET-OX_F	AGGTTCTCGAACCCTAACAACA
RET-OX_R	GCACAGACGACACGTAAGAAAG
CYP81F2_F	GTGAAAGCACTAGGCGAAGC
CYP81F2_R	ATCCGTTCCAGCTAGCATCA
FRK1_F	TTAAACTCGACGATGCAACA
FRK1_R	GATGGAAGTTTTCCCGTTTT
PR1_F	GTAGGTGCTCTTGTTCTTCCC
PR1_R	CACATAATTCCCACGAGGATC
PDF1.2_F	CGCACCGGCAATGGTGG
PDF1.2_R	ATCCATGTTTGGCTCCTTCG
ORA59_F	GGCTCTCGCTTATGATCAGG
ORA59_R	GGACGGTTTCTCATGGAGTG
PR4_F	GTACCACCGCGGACTACTGT
PR4_R	CGTGGAGCAATAAGCACTCA
